# 1-Benzyl-3-phenyl­quinoxalin-2(1*H*)-one

**DOI:** 10.1107/S1600536809039944

**Published:** 2009-10-10

**Authors:** Hanane Benzeid, Nathalie Saffon, Bernard Garrigues, El Mokhtar Essassi, Seik Weng Ng

**Affiliations:** a Laboratoire de Chimie Organique Hétérocyclique, Pôle de compétences Pharmacochimie, Université Mohammed V-Agdal, BP 1014 Avenue Ibn Batout, Rabat, Morocco; b Service Commun Rayons X, Université Paul Sabatier, Bâtiment 2R1, 118 route de Narbonne, 31062 Toulouse, France; c Hétérochimie Fondamentale et Appliquée, Université Paul Sabatier, UMR 5069, 118 Route de Narbonne, 31062 Toulouse, France; d Department of Chemistry, University of Malaya, 50603 Kuala Lumpur, Malaysia

## Abstract

The ten-membered fused ring system in the title compound, C_21_H_16_N_2_O_2_, is planar (r.m.s. deviation = 0.03 Å). The phenyl substituent is aligned at 15.1 (1)° with respect to the mean plane through this system, whereas the phenyl ring of the benzyl substitutent is aligned at 84.4 (1)°.

## Related literature

For the crystal structure of the unsubstituted quinolixalin-2(1*H*)-one, see: Padmaja *et al.* (1987 ▶); Stępień *et al.* (1976 ▶).
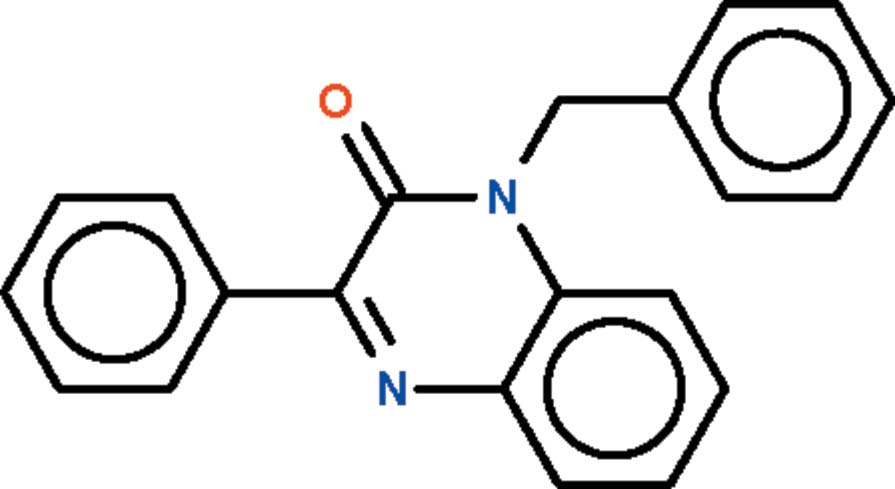


## Experimental

### 

#### Crystal data

C_21_H_16_N_2_O
*M* *_r_* = 312.36
Triclinic, 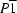

*a* = 5.4776 (2) Å
*b* = 12.7015 (3) Å
*c* = 12.7469 (4) Å
α = 62.260 (2)°
β = 89.963 (2)°
γ = 87.845 (2)°
*V* = 784.23 (4) Å^3^
*Z* = 2
Mo *K*α radiation
μ = 0.08 mm^−1^
*T* = 193 K
0.60 × 0.20 × 0.10 mm

#### Data collection

Bruker APEXII diffractometer
Absorption correction: none
12094 measured reflections
3864 independent reflections
2613 reflections with *I* > 2σ(*I*)
*R*_int_ = 0.037

#### Refinement

*R*[*F* ^2^ > 2σ(*F* ^2^)] = 0.046
*wR*(*F* ^2^) = 0.123
*S* = 1.04
3864 reflections
217 parameters
H-atom parameters constrained
Δρ_max_ = 0.25 e Å^−3^
Δρ_min_ = −0.23 e Å^−3^

### 

Data collection: *APEX2* (Bruker, 2005 ▶); cell refinement: *SAINT* (Bruker, 2005 ▶); data reduction: *SAINT*; program(s) used to solve structure: *SHELXS97* (Sheldrick, 2008 ▶); program(s) used to refine structure: *SHELXL97* (Sheldrick, 2008 ▶); molecular graphics: *X-SEED* (Barbour, 2001 ▶); software used to prepare material for publication: *publCIF* (Westrip, 2009 ▶).

## Supplementary Material

Crystal structure: contains datablocks global, I. DOI: 10.1107/S1600536809039944/tk2547sup1.cif

Structure factors: contains datablocks I. DOI: 10.1107/S1600536809039944/tk2547Isup2.hkl

Additional supplementary materials:  crystallographic information; 3D view; checkCIF report

## Figures and Tables

**Fig. 1.**
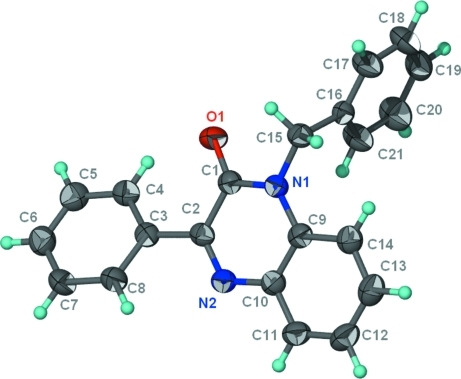
Thermal ellipsoid plot (Barbour, 2001) of C21H16N2O at the 70% probability level; hydrogen atoms are drawn as spheres of arbitrary radius.

## supplementary crystallographic information

### Experimental

To a solution of 3-phenylquinoxalin-2(*1H*)-one (1 g, 4.5 mmol) in *N*,*N*-dimethylformamide (20 ml) was added benzyl chloride (0.62 ml, 5.4 mmol), potassium carbonate (0.74 g; 5.4 mmol) and a catalytic quantity of tetra-n-butylammonium bromide. The mixture was stirred 24 h. The solution was filtered to remove the salts and the solvent then removed under reduced pressure. The residue was recrystallized from ethanol to afford yellow crystals in 85% yield.

### Refinement

Carbon-bound H-atoms were placed in calculated positions (C—H 0.95 to 0.99 Å) and were included in the refinement in the riding model approximation, with *U*_iso_(H) set to 1.2*U*_eq_(C). The amino H-atom was located in a difference Fourier map and was refined with an N–H 0.88±0.01 Å restraint.

### Crystal data

**Table:**

C_21_H_16_N_2_O	*Z* = 2
*M**_r_* = 312.36	*F*(000) = 328
Triclinic, *P*1	*D*_x_ = 1.323 Mg m^−^^3^
Hall symbol: -P 1	Mo *K*α radiation, λ = 0.71073 Å
*a* = 5.4776 (2) Å	Cell parameters from 2441 reflections
*b* = 12.7015 (3) Å	θ = 5.4–28.3°
*c* = 12.7469 (4) Å	µ = 0.08 mm^−^^1^
α = 62.260 (2)°	*T* = 193 K
β = 89.963 (2)°	Plate, yellow
γ = 87.845 (2)°	0.60 × 0.20 × 0.10 mm
*V* = 784.23 (4) Å^3^	

### Data collection

**Table:**

Bruker APEX2 diffractometer	2613 reflections with *I* > 2σ(*I*)
Radiation source: fine-focus sealed tube	*R*_int_ = 0.037
graphite	θ_max_ = 28.3°, θ_min_ = 5.1°
φ and ω scans	*h* = −7→7
12094 measured reflections	*k* = −14→16
3864 independent reflections	*l* = 0→16

### Refinement

**Table:**

Refinement on *F*^2^	Primary atom site location: structure-invariant direct methods
Least-squares matrix: full	Secondary atom site location: difference Fourier map
*R*[*F*^2^ > 2σ(*F*^2^)] = 0.046	Hydrogen site location: inferred from neighbouring sites
*wR*(*F*^2^) = 0.123	H-atom parameters constrained
*S* = 1.04	*w* = 1/[σ^2^(*F*_o_^2^) + (0.0537*P*)^2^ + 0.0846*P*] where *P* = (*F*_o_^2^ + 2*F*_c_^2^)/3
3864 reflections	(Δ/σ)_max_ = 0.001
217 parameters	Δρ_max_ = 0.25 e Å^−^^3^
0 restraints	Δρ_min_ = −0.23 e Å^−^^3^

### Fractional atomic coordinates and isotropic or equivalent isotropic displacement parameters (Å^2^)

**Table:**

	*x*	*y*	*z*	*U*_iso_*/*U*_eq_	
O1	0.8050 (2)	0.89874 (9)	0.53919 (8)	0.0380 (3)	
N1	1.1349 (2)	0.77023 (10)	0.59168 (9)	0.0277 (3)	
N2	0.9967 (2)	0.67563 (10)	0.82777 (10)	0.0305 (3)	
C1	0.9290 (3)	0.81877 (12)	0.61835 (12)	0.0280 (3)	
C2	0.8775 (3)	0.76874 (12)	0.74735 (11)	0.0276 (3)	
C3	0.6910 (3)	0.82891 (12)	0.78827 (12)	0.0286 (3)	
C4	0.4926 (3)	0.89908 (12)	0.71895 (13)	0.0319 (3)	
H4	0.4715	0.9110	0.6402	0.038*	
C5	0.3266 (3)	0.95134 (14)	0.76436 (14)	0.0381 (4)	
H5	0.1920	0.9985	0.7164	0.046*	
C6	0.3539 (3)	0.93598 (14)	0.87820 (14)	0.0407 (4)	
H6	0.2388	0.9722	0.9086	0.049*	
C7	0.5508 (3)	0.86731 (14)	0.94800 (13)	0.0412 (4)	
H7	0.5715	0.8567	1.0264	0.049*	
C8	0.7170 (3)	0.81428 (13)	0.90366 (13)	0.0361 (4)	
H8	0.8510	0.7672	0.9522	0.043*	
C9	1.2618 (3)	0.66951 (12)	0.67667 (12)	0.0289 (3)	
C10	1.1849 (3)	0.62205 (12)	0.79476 (12)	0.0296 (3)	
C11	1.3073 (3)	0.52005 (13)	0.88251 (13)	0.0360 (4)	
H11	1.2555	0.4871	0.9623	0.043*	
C12	1.5020 (3)	0.46731 (14)	0.85381 (14)	0.0408 (4)	
H12	1.5861	0.3990	0.9138	0.049*	
C13	1.5755 (3)	0.51431 (14)	0.73642 (15)	0.0408 (4)	
H13	1.7093	0.4774	0.7169	0.049*	
C14	1.4563 (3)	0.61377 (13)	0.64823 (13)	0.0349 (3)	
H14	1.5065	0.6443	0.5684	0.042*	
C15	1.2157 (3)	0.82914 (12)	0.46821 (12)	0.0303 (3)	
H15A	1.3964	0.8232	0.4678	0.036*	
H15B	1.1655	0.9147	0.4322	0.036*	
C16	1.1145 (2)	0.77719 (12)	0.39268 (12)	0.0274 (3)	
C17	1.1725 (3)	0.82793 (14)	0.27348 (13)	0.0417 (4)	
H17	1.2813	0.8915	0.2418	0.050*	
C18	1.0730 (3)	0.78651 (16)	0.20034 (14)	0.0483 (4)	
H18	1.1141	0.8222	0.1189	0.058*	
C19	0.9165 (3)	0.69502 (15)	0.24384 (15)	0.0445 (4)	
H19	0.8474	0.6678	0.1929	0.053*	
C20	0.8597 (3)	0.64252 (16)	0.36247 (15)	0.0491 (4)	
H20	0.7524	0.5783	0.3938	0.059*	
C21	0.9601 (3)	0.68376 (14)	0.43620 (13)	0.0401 (4)	
H21	0.9213	0.6467	0.5179	0.048*	

### Atomic displacement parameters (Å^2^)

**Table:**

	*U*^11^	*U*^22^	*U*^33^	*U*^12^	*U*^13^	*U*^23^
O1	0.0422 (6)	0.0378 (6)	0.0273 (5)	0.0079 (5)	−0.0037 (5)	−0.0103 (5)
N1	0.0307 (6)	0.0279 (6)	0.0243 (6)	−0.0009 (5)	−0.0005 (5)	−0.0120 (5)
N2	0.0353 (7)	0.0283 (6)	0.0267 (6)	−0.0012 (5)	−0.0012 (5)	−0.0119 (5)
C1	0.0310 (8)	0.0261 (7)	0.0278 (7)	−0.0022 (6)	−0.0023 (6)	−0.0131 (6)
C2	0.0300 (7)	0.0283 (7)	0.0256 (7)	−0.0054 (6)	−0.0005 (6)	−0.0131 (6)
C3	0.0298 (7)	0.0278 (7)	0.0287 (7)	−0.0050 (6)	0.0029 (6)	−0.0132 (6)
C4	0.0305 (8)	0.0334 (8)	0.0321 (7)	−0.0040 (6)	−0.0014 (6)	−0.0153 (6)
C5	0.0300 (8)	0.0397 (8)	0.0421 (9)	−0.0001 (6)	0.0016 (7)	−0.0170 (7)
C6	0.0407 (9)	0.0398 (9)	0.0409 (9)	−0.0013 (7)	0.0113 (7)	−0.0184 (7)
C7	0.0491 (10)	0.0446 (9)	0.0289 (7)	0.0022 (8)	0.0048 (7)	−0.0167 (7)
C8	0.0390 (9)	0.0388 (8)	0.0284 (7)	0.0037 (7)	−0.0009 (6)	−0.0143 (6)
C9	0.0302 (8)	0.0269 (7)	0.0311 (7)	−0.0025 (6)	−0.0047 (6)	−0.0149 (6)
C10	0.0309 (8)	0.0293 (7)	0.0303 (7)	−0.0010 (6)	−0.0041 (6)	−0.0153 (6)
C11	0.0417 (9)	0.0320 (8)	0.0306 (7)	0.0006 (7)	−0.0060 (7)	−0.0115 (6)
C12	0.0408 (9)	0.0324 (8)	0.0447 (9)	0.0068 (7)	−0.0106 (7)	−0.0147 (7)
C13	0.0341 (9)	0.0401 (9)	0.0518 (10)	0.0053 (7)	−0.0034 (7)	−0.0247 (8)
C14	0.0324 (8)	0.0370 (8)	0.0374 (8)	−0.0007 (6)	0.0013 (6)	−0.0192 (7)
C15	0.0319 (8)	0.0298 (7)	0.0275 (7)	−0.0049 (6)	0.0034 (6)	−0.0116 (6)
C16	0.0267 (7)	0.0283 (7)	0.0276 (7)	0.0025 (6)	0.0000 (6)	−0.0136 (6)
C17	0.0480 (10)	0.0441 (9)	0.0312 (8)	−0.0106 (8)	0.0083 (7)	−0.0155 (7)
C18	0.0596 (11)	0.0584 (11)	0.0299 (8)	−0.0017 (9)	0.0045 (8)	−0.0231 (8)
C19	0.0486 (10)	0.0516 (10)	0.0437 (9)	0.0048 (8)	−0.0100 (8)	−0.0316 (8)
C20	0.0533 (11)	0.0530 (10)	0.0497 (10)	−0.0177 (8)	0.0027 (8)	−0.0300 (9)
C21	0.0445 (9)	0.0464 (9)	0.0320 (8)	−0.0139 (7)	0.0068 (7)	−0.0197 (7)

### Geometric parameters (Å, °)

**Table:**

O1—C1	1.2274 (15)	C11—C12	1.376 (2)
N1—C1	1.3820 (18)	C11—H11	0.9500
N1—C9	1.3918 (17)	C12—C13	1.394 (2)
N1—C15	1.4698 (16)	C12—H12	0.9500
N2—C2	1.3000 (17)	C13—C14	1.379 (2)
N2—C10	1.3843 (18)	C13—H13	0.9500
C1—C2	1.4925 (18)	C14—H14	0.9500
C2—C3	1.4872 (19)	C15—C16	1.5132 (18)
C3—C4	1.3971 (19)	C15—H15A	0.9900
C3—C8	1.4008 (19)	C15—H15B	0.9900
C4—C5	1.383 (2)	C16—C21	1.376 (2)
C4—H4	0.9500	C16—C17	1.3887 (19)
C5—C6	1.379 (2)	C17—C18	1.386 (2)
C5—H5	0.9500	C17—H17	0.9500
C6—C7	1.387 (2)	C18—C19	1.366 (3)
C6—H6	0.9500	C18—H18	0.9500
C7—C8	1.382 (2)	C19—C20	1.380 (2)
C7—H7	0.9500	C19—H19	0.9500
C8—H8	0.9500	C20—C21	1.393 (2)
C9—C14	1.396 (2)	C20—H20	0.9500
C9—C10	1.4071 (19)	C21—H21	0.9500
C10—C11	1.4021 (19)		
			
C1—N1—C9	122.23 (11)	C12—C11—H11	119.8
C1—N1—C15	117.13 (11)	C10—C11—H11	119.8
C9—N1—C15	120.63 (11)	C11—C12—C13	119.83 (14)
C2—N2—C10	119.69 (12)	C11—C12—H12	120.1
O1—C1—N1	120.56 (12)	C13—C12—H12	120.1
O1—C1—C2	124.30 (13)	C14—C13—C12	120.87 (15)
N1—C1—C2	115.13 (11)	C14—C13—H13	119.6
N2—C2—C3	117.57 (12)	C12—C13—H13	119.6
N2—C2—C1	122.49 (12)	C13—C14—C9	119.81 (14)
C3—C2—C1	119.89 (11)	C13—C14—H14	120.1
C4—C3—C8	118.12 (13)	C9—C14—H14	120.1
C4—C3—C2	124.19 (12)	N1—C15—C16	113.80 (11)
C8—C3—C2	117.69 (12)	N1—C15—H15A	108.8
C5—C4—C3	120.39 (13)	C16—C15—H15A	108.8
C5—C4—H4	119.8	N1—C15—H15B	108.8
C3—C4—H4	119.8	C16—C15—H15B	108.8
C6—C5—C4	120.92 (14)	H15A—C15—H15B	107.7
C6—C5—H5	119.5	C21—C16—C17	118.26 (13)
C4—C5—H5	119.5	C21—C16—C15	122.73 (12)
C5—C6—C7	119.48 (14)	C17—C16—C15	118.99 (13)
C5—C6—H6	120.3	C18—C17—C16	120.52 (15)
C7—C6—H6	120.3	C18—C17—H17	119.7
C8—C7—C6	120.05 (14)	C16—C17—H17	119.7
C8—C7—H7	120.0	C19—C18—C17	120.85 (15)
C6—C7—H7	120.0	C19—C18—H18	119.6
C7—C8—C3	121.03 (14)	C17—C18—H18	119.6
C7—C8—H8	119.5	C18—C19—C20	119.34 (15)
C3—C8—H8	119.5	C18—C19—H19	120.3
N1—C9—C14	122.46 (13)	C20—C19—H19	120.3
N1—C9—C10	117.81 (12)	C19—C20—C21	119.91 (16)
C14—C9—C10	119.71 (13)	C19—C20—H20	120.0
N2—C10—C11	118.79 (13)	C21—C20—H20	120.0
N2—C10—C9	121.84 (12)	C16—C21—C20	121.11 (14)
C11—C10—C9	119.33 (13)	C16—C21—H21	119.4
C12—C11—C10	120.43 (14)	C20—C21—H21	119.4
			
C9—N1—C1—O1	−171.38 (12)	C2—N2—C10—C11	−178.50 (12)
C15—N1—C1—O1	8.22 (18)	C2—N2—C10—C9	3.8 (2)
C9—N1—C1—C2	9.96 (18)	N1—C9—C10—N2	−2.82 (19)
C15—N1—C1—C2	−170.44 (11)	C14—C9—C10—N2	178.51 (12)
C10—N2—C2—C3	−175.06 (12)	N1—C9—C10—C11	179.47 (12)
C10—N2—C2—C1	2.35 (19)	C14—C9—C10—C11	0.8 (2)
O1—C1—C2—N2	172.36 (13)	N2—C10—C11—C12	−177.24 (13)
N1—C1—C2—N2	−9.05 (18)	C9—C10—C11—C12	0.5 (2)
O1—C1—C2—C3	−10.3 (2)	C10—C11—C12—C13	−1.1 (2)
N1—C1—C2—C3	168.30 (11)	C11—C12—C13—C14	0.4 (2)
N2—C2—C3—C4	−155.97 (13)	C12—C13—C14—C9	1.0 (2)
C1—C2—C3—C4	26.6 (2)	N1—C9—C14—C13	179.85 (13)
N2—C2—C3—C8	24.20 (19)	C10—C9—C14—C13	−1.5 (2)
C1—C2—C3—C8	−153.28 (13)	C1—N1—C15—C16	−92.98 (14)
C8—C3—C4—C5	−0.5 (2)	C9—N1—C15—C16	86.63 (15)
C2—C3—C4—C5	179.64 (13)	N1—C15—C16—C21	0.29 (19)
C3—C4—C5—C6	0.4 (2)	N1—C15—C16—C17	178.13 (13)
C4—C5—C6—C7	0.1 (2)	C21—C16—C17—C18	1.2 (2)
C5—C6—C7—C8	−0.4 (2)	C15—C16—C17—C18	−176.71 (14)
C6—C7—C8—C3	0.2 (2)	C16—C17—C18—C19	−0.1 (3)
C4—C3—C8—C7	0.2 (2)	C17—C18—C19—C20	−0.8 (3)
C2—C3—C8—C7	−179.92 (14)	C18—C19—C20—C21	0.6 (3)
C1—N1—C9—C14	174.04 (12)	C17—C16—C21—C20	−1.4 (2)
C15—N1—C9—C14	−5.54 (19)	C15—C16—C21—C20	176.43 (14)
C1—N1—C9—C10	−4.59 (18)	C19—C20—C21—C16	0.5 (3)
C15—N1—C9—C10	175.83 (12)		
